# Percutaneous Coronary Intervention–Capable Facility Openings and Acute Myocardial Infarction Outcomes by Patient Race and Community Segregation

**DOI:** 10.1001/jamanetworkopen.2023.47311

**Published:** 2023-12-12

**Authors:** Renee Y. Hsia, Yu-Chu Shen

**Affiliations:** 1 Department of Emergency Medicine, University of California, San Francisco; 2 Philip R. Lee Institute for Health Policy Studies, University of California, San Francisco; 3 Department of Defense Management, Naval Postgraduate School, Monterey, California; 4 National Bureau of Economic Research, Cambridge, Massachusetts

## Abstract

This cohort study investigates differential changes in patient outcomes after percutaneous coronary intervention–capable facility openings by patient race and community segregation.

## Introduction

Patients of racial and ethnic minority groups are less likely to be admitted for specialized cardiac care and receive specialized cardiac services compared with White patients.^1,2^ It is possible that these disparities are due to the built environment of where such services exist. We investigated differential changes in patient outcomes after percutaneous coronary intervention–capable facility (PCI-CF) openings by patient race and community segregation.

## Methods

We studied Medicare Fee-for-Service patients with acute myocardial infarction (AMI) from January 1, 2006, to December 31, 2017, and geocoded PCI-CF openings within a 15-minute drive of a community. We divided our patient population into 4 groups based on individual race (Black vs White, administratively identified through the Medicare Master Beneficiary Summary File) and residential segregation (segregated vs integrated, using a dissimilarity index^3^). This retrospective cohort study followed the STROBE reporting guideline and was approved by the National Bureau of Economic Research with no patient consent required because the study used deidentified data.

Statistical analysis was performed from September 1, 2022, to August 20, 2023. We used linear probability models with community fixed effects to determine changes in outcomes (whether the patient received PCI treatment [defined in the eTable in Supplement 1], time-specific mortality) when a community experienced a PCI-CF opening. Analysis was conducted using Stata, version 17.^4^ Results were deemed significant at *P* < .05 using 2-sided tests (eMethods in Supplement 1).^3^

## Results

Of 2 388 180 patients with AMI studied from 2006 to 2017, 27.7% and 63.4% were White patients in segregated and integrated communities, respectively; 4.4% each were Black patients in segregated and integrated communities (Table). For White patients in segregated communities (reference group), the risk-adjusted probability of receiving PCI on day of admission improved by 0.98 (95% CI, 0.19-1.77) percentage points, a 2.1% relative increase, after a PCI-CF opening within a 15-minute drive, relative to that same community type with no PCI capacity change (Figure, A). Black patients in integrated communities showed a 3.92 (95% CI, 2.90-4.95) percentage point increase after a PCI-CF opening, the largest increase of all groups, equivalent to an 10.6% relative increase, which was statistically significantly different from the improvement experienced by the reference group.

**Table.** Descriptive Statistics of Patient Characteristics

Characteristic	Patients, No. (%)			
	Segregated community		Integrated community	
	White (n = 662 343 [27.7])	Black (n = 106 142 ([4.4])	White (n = 1 514 969 [63.4])	Black (n = 104 726 [4.4])
Patient demographic and community characteristics				
Race				
White	662 343 (100.0)	0	1 514 969 (100.0)	0
Black	0	106 142 (100.0)	0	104 726 (100.0)
Sex				
Female	317 257 (47.9)	61 139 (57.6)	703 628 (46.4)	58 099 (55.5)
Male	345 086 (52.1)	45 003 (42.4)	811 341 (53.6)	46 627 (44.5)
Age distribution at time of admission, y				
65-69	122 360 (18.5)	30 174 (28.4)	292 664 (19.3)	30 532 (29.2)
70-74	117 211 (17.7)	21 144 (19.9)	284 309 (18.8)	20 894 (20.0)
75-79	117 235 (17.7)	18 570 (17.5)	277 140 (18.3)	18 349 (17.5)
80-84	120 149 (18.1)	15 955 (15.0)	269 021 (17.8)	15 477 (14.8)
≥85	185 388 (28.0)	20 299 (19.1)	391 835 (25.9)	19 474 (18.6)
Community has high baseline PCI capacity	117 795 (17.8)	21 823 (20.6)	391 312 (25.8)	20 132 (19.2)
Patient conditions				
STEMI	149 241 (22.5)	16 897 (15.9)	342 971 (22.6)	17 339 (16.6)
Peripheral vascular disease	64 388 (9.7)	10 743 (10.1)	152 215 (10.0)	11 133 (10.6)
Pulmonary circulation disorders	32 752 (4.9)	7467 (7.0)	73 569 (4.9)	6674 (6.4)
Diabetes	186 266 (28.1)	41 254 (38.9)	435 613 (28.8)	42 063 (40.2)
Kidney failure	142 692 (21.5)	36 195 (34.1)	323 519 (21.4)	36 093 (34.5)
Liver	5242 (0.8)	1140 (1.1)	12 421 (0.8)	960 (0.9)
Cancer	24 120 (3.6)	4571 (4.3)	54 430 (3.6)	3999 (3.8)
Dementia	28 352 (4.3)	5758 (5.4)	61 906 (4.1)	5682 (5.4)
Valvular disease	90 811 (13.7)	10 973 (10.3)	202 723 (13.4)	11 427 (10.9)
Hypertension	441 048 (66.6)	82 133 (77.4)	1 014 912 (67.0)	81 484 (77.8)
Chronic pulmonary disease	135 533 (20.5)	19 944 (18.8)	322 651 (21.3)	18 971 (18.1)
Rheumatoid arthritis or collagen vascular disease	14 431 (2.2)	1987 (1.9)	34 686 (2.3)	2069 (2.0)
Coagulation deficiency	29 388 (4.4)	5221 (4.9)	65 637 (4.3)	4865 (4.6)
Obesity	44 443 (6.7)	8299 (7.8)	110 635 (7.3)	8855 (8.5)
Substance abuse	7007 (1.1)	2322 (2.2)	18 427 (1.2)	1972 (1.9)
Depression	33 526 (5.1)	2878 (2.7)	73 084 (4.8)	2923 (2.8)
Psychosis	21 406 (3.2)	2715 (2.6)	47 422 (3.1)	2686 (2.6)
Hypothyroidism	76 833 (11.6)	6057 (5.7)	177 887 (11.7)	6167 (5.9)
Paralysis and other neurologic disorder	51 384 (7.8)	10 067 (9.5)	115 074 (7.6)	10 105 (9.6)
Ulcer	1337 (0.2)	271 (0.3)	3092 (0.2)	228 (0.2)
Weight loss	18 916 (2.9)	4588 (4.3)	41 657 (2.7)	4428 (4.2)
Fluid and electrolyte disorders	142 965 (21.6)	27 416 (25.8)	311 734 (20.6)	26 014 (24.8)
Anemia (blood loss and deficiency)	81 514 (12.3)	19 781 (18.6)	188 490 (12.4)	20 046 (19.1)
Treatment and health outcomes				
Same-day PCI	307 892 (46.5)	40 501 (38.2)	702 360 (46.4)	38 726 (37.0)
PCI during hospitalization	411 538 (62.1)	60 755 (57.2)	909 455 (60.0)	56 449 (53.9)
30-d Mortality	87 329 (13.2)	12 195 (11.5)	198 336 (13.1)	12 687 (12.1)
1-y Mortality	187 746 (28.3)	31 212 (29.4)	424 027 (28.0)	31 204 (29.8)

Abbreviations: PCI, percutaneous coronary intervention; STEMI, ST-segment elevation myocardial infarction.

**Figure.**
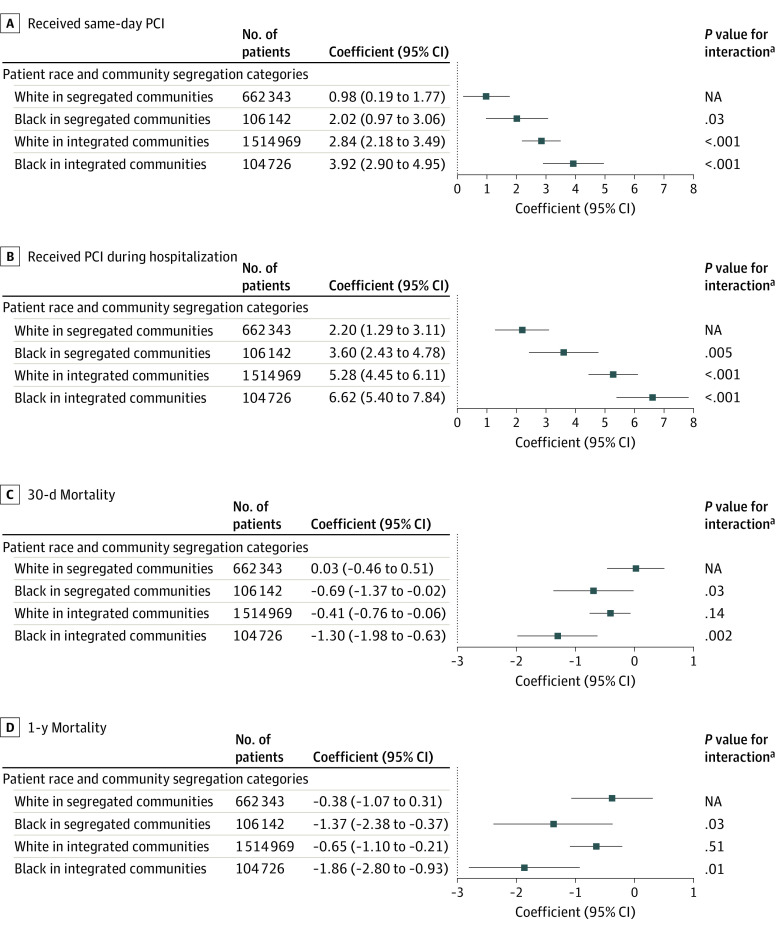
Risk-Adjusted Percentage Point Changes in Outcomes After a Percutaneous Coronary Intervention (PCI)–Capable Facility Opening Within a 15-Minute Drive Range plots indicate 95% CIs of point estimates. NA indicates not applicable. ^a^Testing changes in outcomes that are statistically significantly different from White patients in segregated communities.

Similar patterns were observed in the probability of PCI during hospitalization (Figure, B). Patients in integrated communities had larger increases in their probability of PCI during a hospitalization (6.62 [95% CI, 5.40-7.84] percentage points and 5.28 [95% CI, 4.45-6.11] percentage points for Black and White patients, respectively) than those in segregated communities (3.60 [95% CI, 2.43-4.78] percentage points and 2.20 [95% CI, 1.29-3.11] percentage points for Black and White patients, respectively) after a PCI-CF opening. These changes are equivalent to a 12.3% relative increase for Black patients in integrated communities and a 3.5% relative increase for White patients in segregated communities.

Black patients in integrated communities had a 1.30 (95% CI, –1.98 to –0.63) percentage point decrease, or 10.7% relative decrease in 30-day mortality (Figure, C), and a 1.86 (95% CI, –2.80 to –0.93) percentage point decrease, or 6.2% relative decrease, in 1-year mortality (Figure, D) after a PCI-CF opening, while White patients in segregated communities experienced no statistically significant benefits.

## Discussion

Our study found differential benefits associated with a PCI-CF opening based on patient race and community segregation. Black patients in integrated communities demonstrated the greatest benefits across all outcomes, including a 5 times greater likelihood of receiving same-day PCI after a PCI-CF opening compared with White patients in segregated communities. Study limitations included the use of administrative data and PCI limited to the inpatient setting.

Our findings reveal potential avenues for improving PCI resource allocation, including the implementation of public health measures or financial incentives to support PCI-CF openings in majority Black and integrated communities and greater structural reform targeting the built environment of health care services.

## Notes

Supplement 1. **eMethods.** Additional Methodology (Reproduced From NBER Working Paper #31626)
eReferences.
**eTable.** Complete List of ICD-9 and ICD-10 Procedure Codes Used to Identify Percutaneous Coronary Intervention Treatment

Supplement 2.
Data Sharing Statement
