# Supplementary material for: Percutaneous Coronary Intervention–Capable Facility Openings and Acute Myocardial Infarction Outcomes by Patient Race and Community Segregation
Source: JAMA Netw Open. 2023 Dec 12;6(12):e2347311. doi: 10.1001/jamanetworkopen.2023.47311 (PMC10716732; doi:10.1001/jamanetworkopen.2023.47311)
Supplement: Supplement 1. — eMethods. Additional Methodology (Reproduced From NBER Working Paper #31626) eReferences. eTable. Complete List of ICD-9 and ICD-10 Procedure Codes Used to Identify Percutaneous Coronary Intervention Treatment [file jamanetwopen-e2347311-s001.pdf]

Supplementary Online Content

Hsia RY, Shen YC. Percutaneous coronary intervention–capable facility openings and acute myocardial infarction outcomes by patient race and community segregation. *JAMA Netw Open.* 2023;6(12):e2347311. doi:10.1001/jamanetworkopen.2023.47311

eMethods. Additional Methodology (Reproduced From NBER Working Paper #31626)
eReferences.
eTable. Complete List of ICD-9 and ICD-10 Procedure Codes Used to Identify Percutaneous Coronary Intervention Treatment

This supplementary material has been provided by the authors to give readers additional information about their work.

© 2023 Hsia RY et al. *JAMA Network Open.*

eMethods. Additional Methodology (Reproduced From NBER Working Paper #31626)

Patient Population and Data Sources

Our analytical sample included all Medicare Fee-for-Service patients who were diagnosed with AMI between January 2006 and December 2017. Patients were included in the study population if their primary diagnosis met the following clinical criteria based on the International Classification of Diseases (ICD) codes: 410.x0 or 410.x1 (ICD version 9), or I21 (ICD version 10). In order to get a clean identification of the effects of PCI openings on patient outcomes, our main analysis excluded patients whose communities experienced a PCI closure during the study period. Our control group represented AMI patients in communities that experienced no PCI capacity change during the study period. As our main analysis focused on differences between White and Black patients, we also excluded 5% of the analytical sample that were neither Black nor White.

The primary individual patient data comes from the 100% Medicare Provider and Analysis Review (MedPAR), which contains Medicare beneficiary summary files and is linked to vital status. Relevant data elements include patients' mailing ZIP codes, admission dates, ICD-9 and 10 diagnostic and procedure codes, demographics (e.g., age, sex, race/ethnic group), and date of death. We linked Medicare data with 2010 US Census data via each patient's mailing ZIP code to obtain the longitude and latitude coordinates of their community. This enabled us to construct segregation measures out of the Black and White populations in those communities. In addition, we used the Dartmouth Health Atlas to identify hospital service areas for each ZIP code.[1] To obtain the geographic locations of all hospitals, we used the American Hospital Association (AHA) annual surveys which contain hospital longitude and latitude coordinates, and further supplemented this data with the hospital's heliport coordinates (if existent).[2]

© 2023 Hsia RY et al. *JAMA Network Open.*

Institutional review board approval was not required for this study because no patient-identifiable data was used.

Identifying PCI Facility Openings Over Time

We captured PCI openings within a 15-minute drive of a given community using the following steps. First, for every year we identified whether each hospital was considered PCI-capable using a volume-based approach from prior work in order to minimize self-reporting errors.[3,4] A hospital was considered PCI-capable if it had performed at least 4 PCI procedures (from both inpatient or outpatient settings) in a year. Second, for each hospital, we defined opening year as the first year of at least two consecutive years of operation for a PCI hospital, as done in previous literature.[3,5]

Third, in order to identify changes in PCI capacity for a given community, we first computed actual driving time between each community-hospital pair for every year using web-based maps queries, via HERE developer maps API key and automation software from Stata, based on the geographical coordinates of the pair.[6,7] Finally, having identified the set of PCI-capable hospitals operating within a 15-minute drive for each year, we evaluated year-to-year changes and classified communities according to whether they experienced a PCI hospital opening within a 15-minute drive in a given year. We chose a threshold of 15 minutes based on thresholds reported in other studies,[8–10] and prior literature showing that the majority of hospital visits are within 15 minutes of a patient's residence.[11]

© 2023 Hsia RY et al. *JAMA Network Open.*

Patient Categories Based on Individual Race and Residential Segregation

We categorized our AMI patient population into four categories based on an individual race and community degree of segregation: (1) White in racially segregated communities (reference group); (2) Black in segregated communities; (3) White in integrated communities; and (4) Black in integrated communities. Each individual patient's race was identified from the Medicare beneficiary summary file's race record. Each community's degree of residential segregation was measured at the Hospital Service Area (HSA) level using the dissimilarity index, as residents from ZIP code communities that belonged to the same HSA incurred most of their hospitalizations from hospitals in that area.[12]

The dissimilarity index is the most common measure of segregation that is easy to interpret and has been applied in other health analyses.[13,14] There is a large body of literature devoted to measures of residential segregation.[15–17] The dissimilarity index for the ith HSA was computed as $D_i = \frac{1}{2}\sum_{k=1}^{N}\left|\frac{w_k}{W_i} - \frac{b_k}{B_i}\right|$, where W_i and B_i were the total White and Black population counts at the ith HSA, respectively; and there were N ZIP codes in an HSA, where w_k and b_k were the White and Black population count of the kth ZIP code. We chose ZIP code as the building block of our HSA dissimilarity index based on prior literature.[18,19] HSAs were classified as racially segregated if their White–Black dissimilarity index was in the top one-third of the overall dissimilarity index distribution. Otherwise, they were categorized as integrated. In order to track communities consistently over time, these community measures were made time-invariant and based on 2010 Census data.

Designating Communities with High Baseline PCI Capacity

In one of our sensitivity analyses, we stratified the sample based on a patient's community's baseline PCI capacity. We hypothesized that PCI openings might have a smaller

© 2023 Hsia RY et al. *JAMA Network Open.*

effect on patient health outcomes in communities with high PCI capacity at baseline due to possible duplication of services, and larger effect in communities with low PCI capacity, as the latter communities would have unmet needs.

For the purposes of this analysis, we measured PCI capacity as the percent of patients who were admitted to PCI-capable hospitals (regardless of whether they received PCI) and classified communities as having "high capacity" if they ranked in the top quartile of PCI capacity measures based on their 2005-2006 status. To create a reliable and stable capacity metric, we considered 2 factors: the geographical coverage of each market and the market's PCI capacity. Following prior work,[20–22] we used Hospital Referral Regions (HRR) as the broad market definition to classify communities. The HRR measure accounts for patient flow and transfer patterns and contains a sufficient patient population for obtaining a reliable metric. Similar to prior work,[23] we used the following regression-based approach to rank markets' baseline PCI capacity. Using the 2005 and 2006 AMI population, this risk-adjusted metric was obtained by taking the HRR intercepts from a regression, where the dependent variable was whether the AMI patient was admitted to a hospital with a PCI lab that included separate HRR intercepts on the right-hand side, and controls for patient demographic and comorbid conditions. Rankings based on the HRR intercepts from this regression represented the relative PCI lab capacity for *comparable patient populations* across all HRR markets. For example, if HRR A had a higher coefficient than HRR B, an identical AMI patient would be more likely to have access to a PCI lab in HRR A than in HRR B. We used two years of baseline data to increase the precision of the ranking. ZIP code communities in HRRs ranked in the upper quartile were classified as "high-capacity" markets. In a sensitivity analysis, we used raw PCI capacity to rank

© 2023 Hsia RY et al. *JAMA Network Open*.

the HRRs instead of using the regression-based rank. Our results were robust to the alternate definition.

Statistical Methods

Our analysis focused on changes in the following treatments and health outcomes for AMI patients who experienced a PCI opening within a 15-minute drive of their community: (1) whether the patient received PCI treatment on the day of admission (same-day PCI); (2) whether the patient received PCI treatment during the care episode (PCI during hospitalization); (3) 30-day mortality; and (4) 1-year mortality. Treatments were identified using ICD-9 and ICD-10 procedure codes (detail list in eTable) and procedure dates. In our analysis, we included receipt of coronary angiography in addition to receipt of PCI since this procedure represents a prelude to revascularization and accounts for the clinical realities of failed PCI and/or anatomy that is not suitable for PCI. Mortality outcomes were computed by linking a validated death date with an admission date. We focused on time-specific mortality rather than in-hospital mortality to detect effects on mortality, not only in the acute phase, but in the longer term as well.

Our study design began with a difference-in-differences (DD) framework, where we compared outcomes defined above between patients who experienced a PCI opening within a 15-minute drive from their community (treatment groups) and patients of the same race/segregation category who did not have any change in PCI capacity during the study period (control groups). Take the category of Black patients who lived in segregated communities as an example, our DD framework compares changes in outcomes between a Black patient who lived in a segregated community that experienced a PCI opening and a Black patient who lived in a segregated community that did not experience a PCI opening during the study period. We subsequently implemented a difference-in-differences-in-differences (DDD) framework that allowed us to

© 2023 Hsia RY et al. *JAMA Network Open*.

compare whether the effects of PCI openings on outcomes were statistically significantly different across the 4 patient categories.

Because we had binary outcomes, we estimated a linear probability model with community-fixed effects to control for any unobserved time-invariant heterogeneity across communities and heteroskedasticity-robust standard errors clustered at the community level.[24] We used two sets of key variables. The first set included indicators for whether a community experienced a PCI hospital opening within a 15-minute drive. PCI opening indicators took on a value of 1 on and after the year that a community experienced a PCI opening. The coefficient estimate from this indicator represents changes in outcomes when the reference treatment group (White patients in segregated communities) experienced a PCI opening relative to the control group (patients whose communities did not have a PCI opening during the study period). The second set of key variables included the interaction term between the PCI opening indicators and the patient race/segregation group indicators. The coefficient estimates from this second set represented *additional changes* in outcomes between each race/segregation group relative to the reference group when both groups of patients experienced an opening.

Other control variables in the model included year indicators to capture the macro-level trends, patient demographics (5-year age groups, race and ethnicity, sex), as well as a set of disease-related risk adjustments in accordance with prior work.[25,26] It should be noted that while we controlled for individual race, the race/segregation group indicators and other community-level characteristics were not included in the model, since they were subsumed by the community-fixed effects that already controlled for observed and unobserved differences across communities.

© 2023 Hsia RY et al. *JAMA Network Open.*

We also stratified our analysis based on a community's baseline PCI capacity. This allowed us to examine whether PCI openings had a smaller effect on patient health outcomes in communities with high PCI capacity at baseline, due to the possible duplication of services, and/or if openings had a larger effect in communities with low PCI capacity, as these communities would have unmet needs. As described above, we classified communities as having "high capacity" if they ranked in the top quartile of regression-adjusted PCI capacity measures based on their 2005-2006 status.

© 2023 Hsia RY et al. *JAMA Network Open.*

eReferences.

1. Dartmouth Atlas Project. Dartmouth Atlas Data. Dartmouth Atlas of Health Care. Accessed May 13, 2020. https://atlasdata.dartmouth.edu/static/research_data_archive/?tab=39

2. Horwitz JR, Nichols A. Hospital ownership and medical services: market mix, spillover effects, and nonprofit objectives. *J Health Econ*. 2009;28(5):924-937.

3. Hsia RY, Krumholz H, Shen YC. Evaluation of STEMI Regionalization on Access, Treatment, and Outcomes Among Adults Living in Nonminority and Minority Communities. *JAMA Netw Open*. 2020;3(11):e2025874. doi:10.1001/jamanetworkopen.2020.25874

4. Concannon TW, Nelson J, Goetz J, Griffith JL. A Percutaneous Coronary Intervention Lab in Every Hospital? *Circ Cardiovasc Qual Outcomes*. 2012;5(1):14-20. doi:10.1161/CIRCOUTCOMES.111.963868

5. Baker LC, Phibbs CS. Managed care, technology adoption, and health care: the adoption of neonatal intensive care. *Rand J Econ*. 2002;33(3):524-548.

6. Weber S, Péclat M. A Simple Command to Calculate Travel Distance and Travel Time. *Stata J*. 2017;17(4):962-971. doi:10.1177/1536867X1801700411

7. Developer Guide - HERE Routing API. HERE Developer. Accessed April 30, 2022. https://developer.here.com/documentation/routing-api/dev_guide/index.html

8. Goodman DC, Fisher E, Stukel TA, Chang C. The distance to community medical care and the likelihood of hospitalization: is closer always better? *Am J Public Health*. 1997;87(7):1144-1150.

9. Lam O, Broderick B, Toor S. How far Americans live from the closest hospital differs by community type. Pew Research Center. Accessed December 13, 2021. https://www.pewresearch.org/fact-tank/2018/12/12/how-far-americans-live-from-the-closest-hospital-differs-by-community-type/

10. The Importance of Rural Hospitals. Accessed December 13, 2021. https://ruralhospitals.chqpr.org/Importance.html#fn12

11. Jia P, Wang F, Xierali IM. Differential effects of distance decay on hospital inpatient visits among subpopulations in Florida, USA. *Environ Monit Assess*. 2019;191(2):381. doi:10.1007/s10661-019-7468-2

12. FAQ. Dartmouth Atlas of Health Care. Accessed October 27, 2021. https://www.dartmouthatlas.org/faq/

13. Dimick J, Ruhter J, Sarrazin MV, Birkmeyer JD. Black Patients More Likely Than Whites To Undergo Surgery At Low-Quality Hospitals In Segregated Regions. *Health Aff (Millwood)*. 2013;32(6):1046-1053. doi:10.1377/hlthaff.2011.1365

© 2023 Hsia RY et al. *JAMA Network Open.*

14. Sarrazin MV, Campbell M, Rosenthal GE. Racial Differences In Hospital Use After Acute Myocardial Infarction: Does Residential Segregation Play A Role?: Black Medicare beneficiaries used high-mortality hospitals more often than their white peers, despite their geographic proximity to lower-mortality hospitals. *Health Aff (Millwood)*. 2009;28(Supplement 1):w368-w378. doi:10.1377/hlthaff.28.2.w368

15. Massey DS, Denton NA. The Dimensions of Residential Segregation*. *Soc Forces*. 1988;67(2):281-315. doi:10.1093/sf/67.2.281

16. Massey DS. Reflections on the Dimensions of Segregation. *Soc Forces Sci Medium Soc Study Interpret*. 2012;91(1):39-43. doi:10.1093/sf/sos118

17. Napierala J, Denton N. Measuring Residential Segregation With the ACS: How the Margin of Error Affects the Dissimilarity Index. *Demography*. 2017;54(1):285-309. doi:10.1007/s13524-016-0545-z

18. Agency for Healthcare Research and Quality. Social Determinants of Health Database (Beta Version). Published June 2021. Accessed June 29, 2021. http://www.ahrq.gov/sdoh/data-analytics/sdoh-data.html

19. Sarrazin MSV, Campbell ME, Richardson KK, Rosenthal GE. Racial Segregation and Disparities in Health Care Delivery: Conceptual Model and Empirical Assessment. *Health Serv Res*. 2009;44(4):1424-1444. doi:10.1111/j.1475-6773.2009.00977.x

20. Chandra A, Staiger DO. Productivity Spillovers in Healthcare: Evidence from the Treatment of Heart Attacks. *J Polit Econ*. 2007;115:103-140. doi:10.1086/512249

21. Mohan AV, Fazel R, Huang PH, Shen YC, Howard D. Changes in geographic variation in the use of percutaneous coronary intervention for stable ischemic heart disease after publication of the Clinical Outcomes Utilizing Revascularization and Aggressive Drug Evaluation (COURAGE) trial. *Circ Cardiovasc Qual Outcomes*. 2014;7(1):125-130. doi:10.1161/CIRCOUTCOMES.113.000282

22. Birkmeyer JD, Reames BN, McCulloch P, Carr AJ, Campbell WB, Wennberg JE. Understanding of regional variation in the use of surgery. *Lancet Lond Engl*. 2013;382(9898):1121-1129. doi:10.1016/S0140-6736(13)61215-5

23. Keeley EC, Boura JA, Grines CL. Primary angioplasty versus intravenous thrombolytic therapy for acute myocardial infarction: a quantitative review of 23 randomised trials. *The Lancet*. 2003;361(9351):13-20. doi:10.1016/S0140-6736(03)12113-7

24. Stock JH, Watson MW. Heteroskedasticity-Robust Standard Errors for Fixed Effects Panel Data Regression. :21.

25. Shen YC, Hsia RY. Does decreased access to emergency departments affect patient outcomes? Analysis of acute myocardial infarction population 1996-2005. *Health Serv Res*. 2012;47:188-210. doi:10.1111/j.1475-6773.2011.01319.x

© 2023 Hsia RY et al. *JAMA Network Open.*

26. Skinner J, Staiger D. Technology Diffusion and Productivity Growth in Health Care. *Rev Econ Stat*. 2015;97(5):951-964. doi:10.1162/REST_a_00535

© 2023 Hsia RY et al. *JAMA Network Open*.

eTable. Complete List of ICD-9 and ICD-10 Procedure Codes Used to Identify Percutaneous Coronary Intervention Treatment

Procedure Name	ICD-9 Codes	ICD-10 Codes
Coronary Angiography	3721 3722 3723 8850 8851 8852 8853 8854 8855 8856 8857	4A020N6 4A020N7 4A020N8 4A023N6 4A023N7 4A023N8 B2000ZZ B2001ZZ B200YZZ B2010ZZ B2011ZZ B201YZZ B2040ZZ B2041ZZ B204YZZ B2050ZZ B2051ZZ B205YZZ B2060ZZ B2061ZZ B206YZZ B2100ZZ B2101ZZ B210YZZ B2110ZZ B2111ZZ B211YZZ B2120ZZ B2121ZZ B212YZZ B2130ZZ B2131ZZ B213YZZ B2140ZZ B2141ZZ B214YZZB2150ZZ B2151ZZ B215YZZ B2160ZZ B2161ZZ B216YZZ B2170ZZ B2171ZZ B217YZZ B2180ZZ B2181ZZ B218YZZ B21F0ZZ B21F1ZZ B21FYZZ B5080ZZ B5081ZZ B508YZZ B5090ZZ B5091ZZ B509YZZ B5180ZZ B5181ZZ B518YZZ B5190ZZ B5191ZZ B519YZZ
Percutaneous Coronary Intervention	0066 3600 3601 3602 3604 3605 3606 3607 3609	0270046 027004Z 02700D6 02700DZ 02700T6 02700TZ 0270346 027034Z 02703D6 02703DZ 02703T6 02703TZ 02703Z6 02703ZZ 0270446 027044Z 02704D6 02704DZ 02704T6 02704TZ 02704Z6 02704ZZ 0271046 027104Z 02710D6 02710DZ 02710T6 02710TZ 0271346 027134Z 02713D6 02713DZ 02713T6 02713TZ 02713Z6 02713ZZ 0271446 027144Z 02714D6 02714DZ 02714T6 02714TZ 02714Z6 02714ZZ 0272046 027204Z 02720D6 02720DZ 02720T6 02720TZ 0272346 027234Z 02723D6 02723DZ 02723T6 02723TZ 02723Z6 02723ZZ 0272446 027244Z 02724D6 02724DZ 02724T6 02724TZ 02724Z6 02724ZZ 0273046 027304Z 02730D6 02730DZ 02730T6 02730TZ 0273346 027334Z 02733D6 02733DZ 02733T6 02733TZ 02733Z6 02733ZZ 0273446 027344Z 02734D6 02734DZ 02734T6 02734TZ 02734Z6 02734ZZ 02C03ZZ 02C04ZZ 02C13ZZ 02C14ZZ 02C23ZZ 02C24ZZ 02C33ZZ 02C34ZZ 3E07017 3E070PZ 3E07317 3E073PZ

ICD = International Classification of Diseases.

© 2023 Hsia RY et al. *JAMA Network Open.*
