# Supplementary material for: Percutaneous Coronary Intervention–Capable Facility Openings and Acute Myocardial Infarction Outcomes by Patient Race and Community Segregation
Source: JAMA Netw Open. 2023 Dec 12;6(12):e2347311. doi: 10.1001/jamanetworkopen.2023.47311 (PMC10716732; doi:10.1001/jamanetworkopen.2023.47311)
Supplement: Supplement 2. — Data Sharing Statement [file jamanetwopen-e2347311-s002.pdf]

Data Sharing Statement

Hsia. Percutaneous Coronary Intervention–Capable Facility Openings and Acute Myocardial Infarction Outcomes by Patient Race and Community Segregation. *JAMA Netw Open*. Published December 12, 2023. doi:10.1001/jamanetworkopen.2023.47311

Data
Data available: No
